# Buffalo Academy of Medicine

**Published:** 1908-09

**Authors:** 


### SOCIETY PROCEEDINGS.

### Buffalo Academy of Medicine

**Report of the Subcommittee on Seats and Posture of Children and Stairways.**

The subcommittee has visited a number of schools and has reached the following conclusions: the desks in the schools at present conform very well in general to the types advised by foreign and other authorities; as to the angle of slant of the desk tops; as to the back of the seat projecting forward to fill in the lumbar hollow of the spine; as to the length of the seat; as to the height of the seat; as to the slope of the back of the seat. In many rooms there is one row of desks in which the height of the seat and of the desk top can be adjusted to the child by means of a wrench. In some rooms are small footstools for children with short legs. In one class in one school all the seats were a little too small. In some classes there are naturally several pupils of small size who find the seats too large. Also there are dull, overgrown pupils who find the desks and seats too small. This is provided for partially by having one row of desks of a slightly smaller size in some of the rooms. About 80 per cent. of the pupils were sitting straight even toward the end of a session; the remainder were twisted sideways or leaning on the desk-tops, or bending close to their books, and the like. The teachers seemed to be alive as to the posture question and they must vary individually as to how strongly they enforce the matter.

Some of the stairways were found to be old, and to have the treads badly worn; in many of the older schools the treads are wood and might be replaced by some other material, of an uninflammable nature.

We think there is need of more study of the question; need of frequent inspection by a corps of men who, being paid for their services, would give the subject attention; and need of occasional lectures by these men to the teachers on this and the allied subjects.

(Signed) PRESCOTT LEBRETON,
A. E. WOEHNERT.

### Report of the Subcommittee on Cleanliness.

One of the most important means of maintaining the health of school children is the enforcement of reasonable cleanliness in the school rooms and premises. Such a proposition seems elementary, yet is violated daily in most of the public schools in Buffalo.

The most general and most flagrant violation of ordinary rules of cleanliness is found in the methods of sweeping and dusting. In most of the schools sweeping begins promptly at 3 o'clock, the hour of dismissal of the last classes. All rooms and halls are swept out daily from 3 to 5 o'clock in the afternoon. The sweeping is done with dry brooms and with the windows closed. The mud and dirt that is unavoidably brought into the school, especially in bad weather, is thereby stirred up and much of it is simply swept from the floor into the air as dust. This dust then settles down again upon the floors, desks, chairs, walls, etc., and remains until the next morning, when dusting takes place, between 7 and 8 o'clock. This consists of "*dry dusting*" or stirring up the dust that has settled upon chairs, desks and walls with feather dusters and again diffusing it in the air, where it is ready to injure the health of the children when they assemble in the rooms shortly before 9 o'clock.

The dust evil is the most general, most striking and most easily remediable fault of cleanliness that we have found and we desire to emphasize it and point out how inexcusable and unnecessary this evil is, as is proved by the contrast of the few schools where proper methods of sweeping and dusting are employed.

The floors should be sprinkled with damp sawdust or a substitute before sweeping and dust should be removed from desks, chairs, etc., by wiping with damp cloths.

In some of the schools substitutes for wet sawdust for sprinkling the floors before sweeping have been tried and found most satisfactory. Some of these substitutes are better than sawdust, cheap, non-inflammable, and effective allayers of dust. One of these preparations has been used in Public School No. 36, at a cost of about $1 per month, and with an effectiveness that has won the approval of all concerned.

Many other criticisms relating to cleanliness in the public schools might be made, but we have directed special attention only to the commonest and worst fault. In general it may be said that the cleanliness of any school depends upon its age, construction and intelligence and efficiency of janitor service. These factors vary in the different schools to a considerable extent. The factor that ought to be under better control is the janitor service. Each janitor is responsible to his principal. It is the principal's duty to insist upon proper standards of cleanliness and to report inefficiency of janitor service to the Superintendent of Education, whose power of appointment and removal of janitors in the public schools is absolute. In addition the Board of Health possesses ample power to enforce all proper regulations for making the schools clean and sanitary. Public opinion must find expression and help these public officers to maintain good hygienic conditions in the public schools.

In conclusion, we make the following recommendations:

1. The public ordinance relating to the duties of janitors in the public schools should be amended so as to prohibit dry sweeping and dry dusting as a general daily practice, and to prescribe that wet sawdust or an efficient substitute be used on the floors before sweeping and that all dust be removed from chairs, desks, etc., by damp cloths.

2. Intelligent public opinion should be aroused to hold janitors, principals, the Superintendent of Education, the Health Commissioner, or other officers, responsible for the steady enforcement of proper rules of cleanliness in the public school buildings.

3. A uniform standard of cleanliness should be adopted by the Superintendent of Education and enforced upon the principals and janitors in all schools, instead of allowing, as at present, each one to determine the meaning of cleanliness.

4. The walls and ceilings of rooms and corridors should be painted with oil paints instead of being tinted with water colors, in order to permit thorough washing with soap and water.

5. In the construction of new schools all toilet closets should be placed in extensions from the main building, in order to keep foul odors out of the main building.

6. Boiled (or distilled) water should be provided for all pupils who may desire a safe drinking water or whose parents direct its use. Filtration by ordinary methods does not insure a safe drinking water and should be condemned as a dangerous measure.

(Signed) IRVING P. LYON,
MAUDE J. FRYE.

**Report of Subcommittee on Ventilation and Heating.**

The public school buildings of this city are variously ventilated and heated. In the more recently constructed buildings a fan located in the basement and operated by a steam engine draws in the air from outside and then forces it through the various classrooms and out again at the roof. This is called the plenum system. In the very latest buildings presenting this system the heating is accomplished mainly by what is called the direct method, that is, by means of steam radiators placed in the various class rooms and halls. The air forced in is simply for the purpose of ventilation. It is not relied on to heat the building. It is warmed to a temperature of about 70°F.

In many school buildings, however, having the plenum system of ventilation, the heated air blown in is the only source of heat for the building. Since in this method the heating of the air is not accomplished directly in the separate rooms, but at a distance in the basement it is called the indirect system. In this indirect hot air system the air chamber in the basement is divided so that a portion of the air passing over steam coils is heated to about 60°F; while the remainder passes over a large number of coils and is heated to about 120°F. Each class room is connected by air ducts in such a way that a thermostat located in the room controls valves and dampers and determines whether air at 60°F or at 120°F enters the room. The heating of the room with this method depends upon the amount and the temperature of the air blown in.

In many of the school buildings the vacuum system so-called is in operation. Here the fan is employed to draw the air out of the class rooms. In this system the fan is generally in the attic, located in a chamber into which empty the air ducts from the various rooms. The fresh air enters at several openings in the basement and passes over steam coils to be warmed to a proper temperature before entering the class rooms. With this system of ventilation we may have either direct or indirect heating.

In some school buildings an attempt has been made to combine the plenum and the vacuum systems by placing fans both in the basement and in the attic—one to blow air in, the other to draw it out. There are also numerous schools in which there is no mechanical device for creating air currents. These present two classes. In the first, the heating of the air is relied upon to create a current. The hot air furnace exemplifies this type. Because of the difference in quantity of air at different temperatures this is often called the gravity system. In the second variety no artificial heat is utilised and the air current in the air shafts, if any, depends upon the difference in the temperatures of the outside air and of that within the buildings. This method has been called the natural system. Finally, there are school buildings where windows are practically the only reliance for ventilation. In some instances deflectors for preventing direct draughts are provided. In other cases these are lacking.

During the last few years, several examinations have been made, under the auspices of the Department of Health to determine the amount of air furnished; its diffusion in the class rooms and the degree of its contamination. The reports of these examinations are on file at the office of the department.

Of all the schools thus examined only two have shown perfect ventilation. These are the Lafayette High School and the new No. 24 Grammar School. The ventilating systems of both of these schools were planned by and installed under the supervision of Mr. Woodbridge, a sanitary engineer connected with the Massachusetts School of Technology. These two instances prove that a system of ventilation can be devised and installed which will give adequate ventilation. In nearly all the other schools of this city the ventilation is very seriously inadequate; and what adds to the difficulty is that attempts to supplement this inadequate ventilation by opening windows disarranges the mechanical systems and often makes matters worse. The reason for this is clear when it is understood that in the plenum system, for example, the air is forced into a chamber from which it is carried to the various class rooms by means of air ducts. The air being under pressure it naturally goes where there is the least resistance. If one or more windows are opened in one of the class rooms a disproportionate amount of air escapes by way of this room, and this at the expense of the rest of the rooms. Therefore the rule that windows must be kept closed. But when the amount of air furnished by the fan system is inadequate, and when also perhaps its diffusion about the room is imperfect, the result is a serious harm to the well being and health of the teacher and children.

The expense of these ventilating plants is large. In view of their very general inadequacy and the serious consequences to health, a decided change is demanded. Either the city should employ a thoroughly competent and honest sanitary engineer to see to it that the plans are scientifically correct and that the installation is in honest accord with the plans, or the pretense should be stopped, and all mechanical systems be abolished.

Reliance upon open windows with some devices for deflecting draughts and perhaps for warming the entering air would be far better than the present costly inadequate and health destroying systems now almost universal in our schools. Want of scientific understanding is seen all about, and the examples of positive dishonesty have not been few in the past.

Meanwhile the condition may be much improved by a general opening of doors and windows for a few minutes at least once during each morning and afternoon session. This periodical airing with contemporaneous calisthenic exercise we strongly advise and urge.

The heating of the public school buildings is in general fairly satisfactory. The best system in use is that found in the later schools—namely, direct heating by steam coils. In the schools heated by hot air blown in by a fan and controlled by thermostats the sudden change from air at 120° F to that at 60° F, or vice versa is unpleasant as well as detrimental to children sitting in line with the column of entering air. Other objections to this method are that to heat a single room involves operating the engine and fan and that a failure of either means inability to heat the building.

Stoves have practically disappeared except in a few of the annexes.

(Signed) P. W. Van Peyma.

Max Keiser.

H. K. De Groat.

**Report of the Committee on Lighting and Shades.**

In the main these conditions were found to be very good, excepting for the teachers and left-handed pupils. Generally, the light comes from windows on the left of the pupils which is as it should be for those who are right-handed; but some rooms have windows in the back end, which are very trying to the eyes of the teacher.

There are from two or three to a dozen left-handed pupils in each school, and for these, the lighting arrangements are exactly the reverse of what is proper.

We would recommend:

1. That windows in the ends of rooms, facing either teachers or pupils be closed in every instance when sufficient light is obtainable from side.

2. That all left-handed pupils be supplied with desks facing in the opposite direction from the others, so the light will come from their right sides. This may easily be arranged.

3. That all shades be of the adjustable type, admitting light both above and below, dark yellow or light green in color and so arranged as to distribute the light as evenly as possible throughout the room.

4 That all ceilings be tinted a light yellow and all side walls be painted a medium green shade and without luster because it is important to avoid reflections.

(Signed) L. BURROWS, JR.
JOHN R. GRAY.

